# Cognitive and brain reserve predict decline in adverse driving behaviors among cognitively normal older adults

**DOI:** 10.3389/fpsyg.2022.1076735

**Published:** 2022-12-23

**Authors:** Samantha A. Murphy, Ling Chen, Jason M. Doherty, Prerana Acharyya, Noah Riley, Ann M. Johnson, Alexis Walker, Hailee Domash, Maren Jorgensen, Sayeh Bayat, David B. Carr, Beau M. Ances, Ganesh M. Babulal

**Affiliations:** ^1^Department of Neurology, Washington University School of Medicine, St. Louis, MO, United States; ^2^Division of Biostatistics, Washington University School of Medicine, St. Louis, MO, United States; ^3^Center for Clinical Studies, Washington University School of Medicine, St. Louis, MO, United States; ^4^Department of Biomedical Engineering, Schulich School of Engineering, University of Calgary, Calgary, AB, Canada; ^5^Department of Geomatics Engineering, Schulich School of Engineering, University of Calgary, Calgary, AB, Canada,; ^6^Department of Medicine, Division of Geriatrics and Nutritional Science, Washington University School of Medicine, St. Louis, MO, United States; ^7^Hope Center for Neurological Disorders, Washington University School of Medicine, St. Louis, MO, United States; ^8^Washington University School of Medicine, Mallinckrodt Institute of Radiology, St. Louis, MO, United States; ^9^Washington University School of Medicine, Institute for Public Health, St. Louis, MO, United States; ^10^Department of Psychology, Faculty of Humanities, University of Johannesburg, Johannesburg, South Africa; ^11^Department of Clinical Research and Leadership, The George Washington University School of Medicine and Health Sciences, Washington, WA, United States

**Keywords:** cognitive reserve, brain reserve, driving (veh), aging, older (elderly) drivers

## Abstract

Daily driving is a multi-faceted, real-world, behavioral measure of cognitive functioning requiring multiple cognitive domains working synergistically to complete this instrumental activity of daily living. As the global population of older adult continues to grow, motor vehicle crashes become more frequent among this demographic. Cognitive reserve (CR) is the brain’s adaptability or functional robustness despite damage, while brain reserve (BR) refers the structural, neuroanatomical resources. This study examined whether CR and BR predicted changes in adverse driving behaviors in cognitively normal older adults. Cognitively normal older adults (Clinical Dementia Rating 0) were enrolled from longitudinal studies at the Knight Alzheimer’s Disease Research Center at Washington University. Participants (*n* = 186) were ≥65 years of age, required to have Magnetic Resonance Imaging (MRI) data, neuropsychological testing data, and at least one full year of naturalistic driving data prior to the beginning of COVID-19 lockdown in the United States (March 2020) as measured by Driving Real World In-vehicle Evaluation System (DRIVES). Findings suggest numerous changes in driving behaviors over time were predicted by increased hippocampal and whole brain atrophy, as well as lower CR scores as proxied by the Wide Range Achievement Test 4. These changes indicate that those with lower BR and CR are more likely to reduce their driving exposure and limit trips as they age and may be more likely to avoid highways where speeding and aggressive maneuvers frequently occur.

## 1. Introduction

Motor vehicle crashes are the eight leading causes of death globally, across all age groups (Rosen et al., 2022). In the United States, motor vehicle crashes are the second leading cause of death from unintentional injuries among adults aged 65+ (Rosen et al., 2022). Driving is the ultimate expression of functioning requiring multiple domains working concertedly (sensory, motor, visual, cognitive processes) to complete this complex instrumental activity of daily living (IADL; Babulal, 2022). The risk of a crash, decline in health, or family concerns may influence the decision to retire from driving (Campbell et al., 1993; O’neill et al., 2000). Driving retirement may reduce the risk of crashes and resulting fatalities; however, this creates an elevated risk of psychosocial sequalae such as depression, isolation, and admission to institutional care (Freeman et al., 2006; Edwards et al., 2009; Qin et al., 2020). To reduce the risk of motor vehicle crash fatalities without increasing driving retirement, some older adults may begin to regulate driving behaviors to maintain their independence. This may include trip reduction, driving at specific times, or avoiding highways/high-traffic roads. Due to an anticipated growth of older adult drivers over the next three decades, it is more important to consider options to preserve driver safety and monitor decline in driving behavior (Pollack, 2005).

Cognitive reserve (CR) and brain reserve (BR) are promising constructs posited to explain why differences in aging outcomes persist in the presence of cognitive decline or neurological diseases like dementia. CR is considered to be the breadth of memory and thinking abilities, including flexibility and adaptability of attentional processes (conscious and subconscious) commonly subserving daily function (i.e., work, play, and leisure; Stern et al., 2020). Conversely, BR refers to physical and neuroanatomical structures of the brain (Stern et al., 2020). More simply, CR can be conceptualized as the brain’s software, while BR is the hardware, both functioning synergistically to support human function. Since BR can be quantitatively measured *via* brain volume or neuronal count, it is often simpler to compare BR values than CR in aging studies. CR measurement relies on common proxies (e.g., education, literacy, and occupational attainment) that evaluates sociobehavioral components which may contribute to individual reserve (Tucker and Stern, 2011; Stern et al., 2020). However, these proxies are often conducted *via* collateral source or self-report and may not allow for the objective investigation of CR’s impact on complex tasks such as driving.

Driving provides a novel medium to examine the breadth and depth of cognitive functioning. In a study examining IADL and cognition, better scores on timed measures were associated with increased scores on the Mattis Dementia Rating Scale-Second Edition, while increased awareness of self-reported IADL performance was also associated with higher levels of CR (Suchy et al., 2011). Older adults with high CR seem to have an increased awareness of their deficits and can implement compensatory strategies early, which may be effective during more serious decline (Suchy et al., 2011). A recent study from Milan, Italy assessed healthy, community-residing older adults’ fitness to drive and found that higher CR (education as proxy) was associated with improved driving skills such as processing speed and decision speed when assessing cognitively normal older adults’ fitness to drive (Balzarotti et al., 2021). Another study that investigated age-related decline on driving *via* a driving simulator demonstrated that older adults with higher scores on a cognitive battery assessing logical reasoning, spatial visualization, processing speed, executive control, and perceptual-motor skills were able to alter their speed and time-to-stop (headway) more quickly (Andrews and Westerman, 2012). One proposed explanation is that older adults may strategically compensate, selecting driving conditions and parameters that reduce task demands, such as reducing night driving or avoiding heavy traffic areas. Since awareness of deficits is associated with CR level, performance in daily living tasks may also be impacted by CR. However, generalizability to daily driving behavior is limited since the extant literature assessed how simulated driving and road testing are impacted by cognitive reserve. There is a significant gap in the literature that examines complex human function (i.e., IADLs) and CR/BR beyond prototypical neuropsychological tasks. Additionally, prior studies have sought to determine differences in cognitive function that may contribute to adverse driving behaviors among cognitively normal older adults with and without preclinical Alzheimer’s disease and found no significant difference (Roe et al., 2017a,b). Furthermore, research has investigated associations between several cognitive substrates and driving decline in relation to driving behavior and have found unreliable relationships (Aschenbrenner et al., 2022). Therefore, the present study seeks to fill a gap in literature that is necessary to better understand if CR and BR constructs contribute to changes of the complex IADL of driving. The present study chose not to propose mechanisms that could impact driving ability since naturalistic driving is a novel and highly variable task that is measured in real time with unpredictable events that influence driver response.

Recent advances in telematics and global position systems (GPS) technology have produced affordable and unobtrusive dataloggers that allow tracking of individual vehicles and the acquisition of daily driving data *via* velocity, spatial, and temporal characteristics (Babulal et al., 2016). In this study, we investigate the relationship between CR, vis-a-vie a reading fluency task and longitudinal naturalistic driving behaviors measured *via* an in-vehicle data logger. We also investigate the relationship between daily driving and BR evaluated by hippocampal and whole brain volume quantified *via* magnetic resonance imaging (MRI). We hypothesize that older drivers with lower CR and BR at baseline will demonstrate less self-regulation (e.g., lower miles driven) and have an increase in adverse driving behavior over time, such as over speeding and hard braking.

## 2. Materials and methods

### 2.1. Participants

Participants were enrolled in longitudinal aging and driving studies at the Knight Alzheimer’s Disease Research Center (ADRC) at Washington University School of Medicine. Participants included in this study were at least 65 years of age or older, were cognitively normal at baseline as evaluated by a 0 on the Clinical Dementia Rating (CDR®; Morris, 1993), and did not progress upon subsequent follow-up assessment. Additionally, participants had their magnetic resonance imaging (MRI) within 2 years of enrollment in the longitudinal driving study. Participants self-reported driving at least once per week and had naturalistic driving data available for at least for one full year to account for seasonality and daylight time changes. Additionally, longitudinal methods were implemented to investigate intraindividual change over time as one continues to drive. All data were limited to 3/31/2020 to exclude the effects of pandemic guidelines (e.g., lockdown, restrictions, shelter-in-place) on driving behavior (Roe et al., 2021). Additionally, all participants either self-identified as white or Black for race.

### 2.2. Naturalistic driving

A datalogger (Azuga G2 Tracking device) was installed into each participant’s vehicle’s onboard diagnostic port (OBDII). Driving behavior was continuously collected and for a given “trip,” defined as ignition start to ignition off, data collected includes the date, time, vehicle speed, latitude and longitude, and adverse events such as speeding, hard braking, and sudden acceleration. We have used the Driving Real-World In-Vehicle Evaluation System (DRIVES; Babulal et al., 2016, 2019; Roe et al., 2019) to examine various metrics, including the total number of trips, the average distance traveled, number of night trips taken, number of trips across multiple distances from their home, idle time, over speeding, hard braking, and sudden acceleration. Idle time reflects the amount of time a vehicle is started in park but not moving. Over speeding events are defined as a speed six MPH or more over the posted speed limit. Hard braking is defined as a sudden decrease in speed of eight MPH or more per second. Sudden acceleration defined as an increase in speed of eight MPH or more per second. Over speeding, hard braking, and speeding were conceptualized as adverse driving behavior.

### 2.3. Cognitive reserve

The Word Reading subtest (the blue form) of the Wide Range Achievement Test 4 (WRAT 4) served as a proxy for cognitive reserve (Tucker and Stern, 2011; Barulli and Stern, 2013). The Word Reading subtest is administered annually during an office testing session. In the subtest, the participant is asked to read 55 words aloud to the administrator. The participant reads until they have incorrectly pronounced 10 words consecutively at which point the task is discontinued or have finished the list (Wilkinson and Robertson, 2006). WRAT 4 scores were compared *via* dichotomized groups with those performing amongst the top two thirds of the sample being considered high performers and those performing amongst the bottom third being considered poor performers. The WRAT Reading subtest has been utilized, validated, and commonly used as a proxy for CR amongst older adults in previous research (Siedlecki et al., 2009; Brickman et al., 2011; Baker et al., 2017).

### 2.4. Brain reserve

An MRI was completed on a 3 T Tesla using a research imaging protocol that is based upon the Alzheimer’s Disease Neuroimaging Initiative (ADNI), which includes a high-resolution T1 MPRAGE for assessment of brain structures to produce normalized whole brain volume (WBV) and hippocampal volume (HV) measurements. Prior to analysis, HV was normalized to account for differences in head size. The procedure consisted of computing the mean intracranial volume (ICV) for the sample, and then conducting a regression analysis with ICV as the sole independent variable and participants’ HV (the sum of right and left HV) as the dependent variable. The β-weight was then used to compute participants’ normalized HV using the following equation: normalized HV = raw HV – [β-weight × (participant’s ICV – sample mean ICV); Buckner et al., 2004]. These elements are part of standard clinical brain MRIs. All MRIs were reviewed by board-certified neuroradiologists. WBV and HV were determined by the neuroradiologist based on standard clinical assessment. Details of the structural brain MRI and radiological assessment are available in prior publications (Koenig et al., 2021, 2022). WBV and HV were each assessed dichotomously, comparing the top two thirds with the bottom third where the lower group represented increased atrophy.

### 2.5. Cognitive assessment

All participants were administered an annual comprehensive clinical and cognitive assessment. A trained clinician rated the participant for presence and severity of dementia symptoms using the CDR® (Morris, 1993). The cognitive battery consists of several assessments of cognitive functioning and included the following assessments: the free recall test from the Free and Cued Selective Reminding test (Grober et al., 1988) to measure episodic memory; Trail Making Parts A and B (Armitage, 1946) to measure processing speed, and Animal Naming (Tombaugh et al., 1999) to measure semantic fluency. Each cognitive test was z-scored to the baseline cognitive assessment, defined as the cognitive assessment nearest a participant’s enrollment into the driving study, and were averaged to form a composite score.

### 2.6. Statistical analysis

The reserve markers were dichotomized using the first tertile and the top two tertiles to classify participants into lower/higher risk groups, respectively. Descriptive statistics summarized key demographics variables and compared risk groups using independent t-test or χ^2^ test, McNemar’s test assessed the concordance of the three markers for classifying participants into lower/higher risk. Longitudinal data analyses assumed a linear relationship between aggregated monthly naturalistic driving variables and the markers. A random coefficients model (linear mixed model) was used to predict the average rate of change in the driving outcomes based on the groups adjusting for age (centered at the sample mean), education, and gender. This model allowed y-intercepts and slopes (monthly rate of change) to vary randomly between participants and fitted a separate regression line for each participant. The y-intercept generated from the linear mixed model estimated the mean of a driving variable at the beginning of data collection (baseline). The interaction between marker group and time was considered for testing for slope difference over time, and the model examined whether there was a difference at the y-intercept. Predicted means of the driving variables of each participant were obtained from the random coefficients model, and locally weighted scatter plot smoothing was applied to visualize the estimated y-intercept and slope change over time between groups. Sensitivity analysis was conducted to determine if accounting for education would impact the slope models. Akaike information criterion (AIC) as a model fit statistics was compared between the random coefficients model with and without including education. The random coefficients model including education yielded smaller AIC and thus was selected. All statistical analyses were two-tailed at a significance level of 0.05 and performed with SAS 9.4 (SAS Institute, Cary, NC).

## 3. Results

A total of 186 older adults met inclusion criteria. On average, participants were in their early 70’s, well-educated, non-Hispanic white, and had similar sex distribution (Table 1). Longitudinal driving was present for over 4 years with a mean centering around 2 years. The WRAT was moderately correlated with CR (*r* = 0.52; *p* = <0.0001) but not the two measures of BR (HV [*r* = −0.07; *p* = 0.3009] WBV [*r* = 0.02; *p* = 0.7264]). There was a moderate correlation between CR and BR (*r* = 0.61; *p* = <0.0001). There were no statistically significant differences between males and females found for the WRAT (*p* = 0.7385), HV (*p* = 0.3155), and WBV (*p* = 0.6774). Two-sample t-test showed that no significant differences across age for high and low performers on the WRAT (*p* = 0.9367). However, a statistically significant difference was found across age between those with lower and higher values of HV (*p* < 0.0001) and WBV (*p* < 0.0001). Significant differences were found for education level between high and low performers on the WRAT (*p* < 0.0001), as well as those with low and high values of HV (*p* = 0.0258). Lastly, statistically significant differences for cognitive composite score were found for those with low and high values of only WBV (*p* = 0.0146) but not for WRAT 4 (*p* = 0.0844) or HV (*p* = 0.1493). The random coefficient models were conducted in which numerous slope models for driving metrics were found to be statistically significant (Table 2).

**Table 1:** Baseline demographics (*N* = 186)*.

	Sample	WRAT 4		WBV		HV	
		+	-	+	-	+	-
Age (years)	73.77 ± 4.98	73.64 ± 4.92	73.70 ± 5.12	75.93 ± 5.92	72.54 ± 4.49^***^	76.39 ± 5.10	72.34 ± 4.37^**^
Education (years)	16.66 ± 2.21	17.48 ± 1.88	15.28 ± 2.03^***^	16.57 ± 2.45	16.71 ± 2.08	17.20 ± 2.33	16.40 ± 2.10^*^
Women, N (%)	100 (53.76%)	64 (34.41%)	36 (19.35%)	68 (36.56%)	32 (17.20%)	64 (34.41%)	36 (19.35%)
Race, Caucasian, N (%)	160 (86.02%)	108 (58.06%)	52 (27.96%)^**^	109 (58.60%)	51 (27.42%)	108 (58.06%)	52 (27.96%)
Follow up time (years)	2.16 ± 0.79	2.12 ± 0.80	2.23 ± 0.77	2.10 ± 0.82	2.19 ± 0.77	2.04 ± 0.84	2.22 ± 0.76
Cognitive composite (z-score)	−3.23E-11 ± 0.7197	0.08 ± 0.64	−0.13 ± 0.82	−0.19 ± 0.78	0.10 ± 0.67^*^	−0.11 ± 0.76	0.06 ± 0.15
WRAT 4, Low Performers, N (%)	69 (37.10%)						
WBV, Increased Atrophy, N (%)	62 (33.33%)						
HV, Increased Atrophy, N (%)	61 (32.80%)						

Mean ± Standard Deviation or count (percentage). Note: WRAT 4 (Cognitive Reserve), WBV (Whole brain volume, Brain Reserve), HV (Hippocampal volume, Brain Reserve). The (+) slope values refer to increased volume of brain structure in the Whole Brain Volume and Hippocampal Volume (Brain Reserve) sections as well as higher performance in the WRAT 4 (Cognitive Reserve) section. While the (−) slope values refer to increased atrophy of brain structure and poorer performance on the WRAT 4.

*** *p* < 0.0001.

** *p* < 0.001;

* *p* < 0.05.

**Table 2:** Change over time as predicted by slopes of daily driving outcomes by three groups.

Slopes					
	Mean	SE	Mean	SE	*p*
WRAT 4					
Idle Time	0.5063	0.1604	0.3888	0.2010	0.0012
*Overspeeding	−0.0010	0.0003	−0.0007	0.0004	0.0035
*Hard Braking	−0.0001	0.0005	−0.0017	0.0006	0.0180
*Adverse	−0.0009	0.0003	−0.0011	0.0004	0.0024
Day Trips	−0.3057	0.0779	−0.3323	0.0972	< 0.0001
Night Trips	0.3191	0.1020	0.3517	0.1283	0.0004
Mixed trips	0.0052	0.0050	0.0094	0.0040	0.0429
Whole Brain Volume					
Idle Time	0.9179	0.2137	0.2637	0.1454	< 0.0001
*Overspeeding	−0.0394	0.0144	−0.0100	0.0099	0.0153
*Hard Braking	−0.0007	0.0006	−0.0007	0.0004	0.1868
*Adverse	−0.0018	0.0004	−0.0005	0.0003	< 0.0001
Day Trips	−0.4393	0.1134	−0.2724	0.0771	< 0.0001
Night Trips	0.2018	0.1436	0.4036	0.0985	0.0002
Mixed trips	0.0051	0.0056	0.0089	0.0038	0.0481
Hippocampal Volume					
Idle Time	0.6104	0.2215	0.4087	0.1439	0.0005
*Overspeeding	−0.0010	0.0004	−0.0008	0.0003	0.0035
*Hard Braking	−0.0005	0.0006	−0.0008	0.0004	0.1719
*Adverse	−0.0011	0.0005	−0.0009	0.0003	0.0016
Day Trips	−0.3187	0.1132	−0.3176	0.0735	< 0.0001
Night Trips	0.3990	0.1478	0.3115	0.0972	0.0003
Mixed trips	0.0095	0.0057	0.0069	0.0037	0.0455

The (+) slope values refer to increased volume of brain structure in the Whole Brain Volume and Hippocampal Volume (Brain Reserve) sections as well as higher performance in the WRAT 4 (Cognitive Reserve) section. While the (−) slope values refer to increased atrophy of brain structure and poorer performance on the WRAT 4.

* Each variable represents the mean number per trip.

### 3.1. Driving behavior

**Figure 1:**
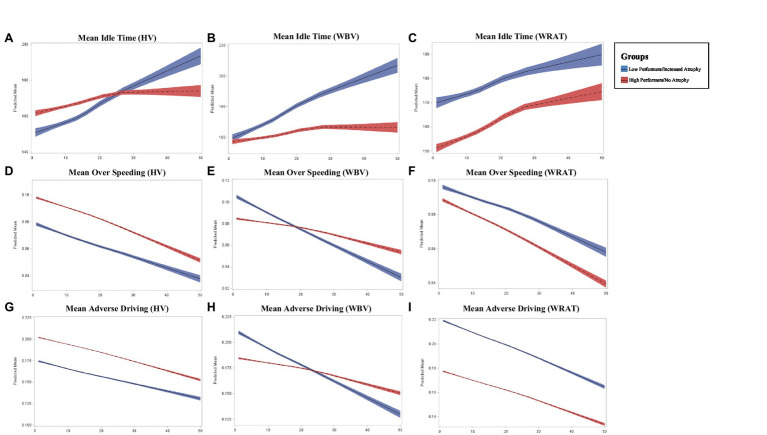
Longitudinal change in adverse driving outcomes across groups where *x*-axis represents the number of months (0–50) and *y*-axis represents the predicted mean in driving outcome **(A–I)**.

Lower HV and WBV were found to predict increased idle time across follow-up (*p* = 0.0005; *p* < 0.0001), compared to those without atrophy (Figure 1). Additionally, lower performers on the WRAT 4 were also found to have increased idle time (*p* = 0.0012) compared to high performers. Participants with lower HV and WBV experienced a significant decrease in over speeding at a greater rate of change compared to those without HV or normalized WBV atrophy (*p* = 0.0035; *p* = 0.0003). Poor performers on the WRAT 4 also showed a significant decrease in over speeding compared to high performers (*p* = 0.0035). Poor performers on the WRAT 4 showed a statistically significant decrease in hard braking over time (*p* = 0.0180), while WRAT 4 high performers showed little to no change in hard braking behavior over time. Comparatively, decreased HV and WBV were found to predict significant decreases in adverse driving behaviors over time (*p* = 0.0016; *p* < 0.0001). Those with decreased HV and WBV show a greater change longitudinally. The same was found for poor performers on the WRAT 4 (*p* = 0.0024).

### 3.2. Driving frequency

**Figure 2:**
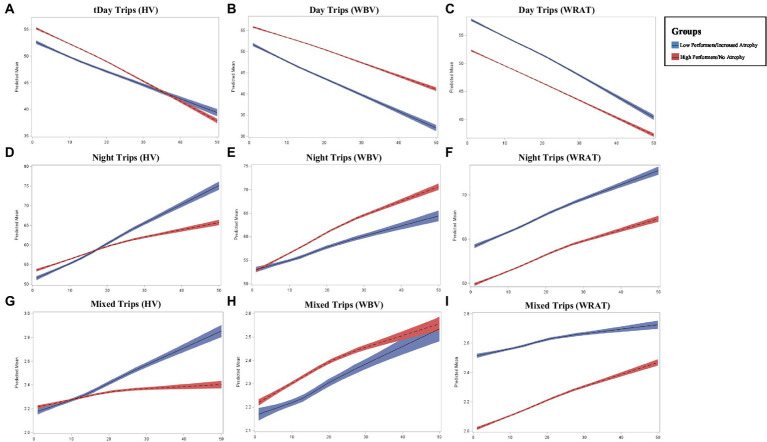
Longitudinal changed in driving frequency outcomes across groups where *x*-axis represents the number of months (0–50) and *y*-axis represents the predicted mean in driving outcome **(A–I)**.

While both high and low performers across HV and WBV showed a decrease in trips taken during the day, those with an absence of atrophy showed a steeper decline in daytime trips over time (*p* < 0.0001; *p* < 0.0001; Figure 2). For WRAT 4 high performers, a steeper decline in daytime trips overtime was also found (*p* < 0.0001). Conversely, those with increased atrophy in HV and WBV showed a steeper increase in nighttime trips over time (*p* = 0.0003; *p* = 0.0002) compared to those with lower levels of atrophy. A steeper increase in nighttime trips overtime was also found (*p* = 0.0004) for poor performers on the WRAT 4. Although both groups across HV and WBV showed an increase in trips taken daily with variability in start and end time, those with increased atrophy show a more significant increase in variability (*p* = 0.0455; *p* = 0.0481). For poor performers on the WRAT 4, a steeper increase in variability of start and end time of trips was found (*p* = 0.0429).

## 4. Discussion

We investigated the relationship between CR performance as proxied by the WRAT 4 and naturalistic driving behavior, assessing a complex IADL as measured *via* an in-vehicle data logger. We also investigated the relationship between BR evaluated by HV and WBV and daily driving. Numerous changes in daily driving behaviors over an average of 2.15 years were predicted by decreased HV and WBV, and lower CR scores proxied by the WRAT 4. Due to the lack of statistical significance on the cognitive composite with performance on the WRAT, these behavioral changes cannot be explained by cognitive performance resulting from subsequent impairments. Longitudinal changes in daily driving suggests that cognitively normal older adults regulate their driving over time. Those with lower BR and CR are more likely to restrict their driving behavior and adapt their daily behaviors such as trips made during the day, speeding, and hard braking as time progresses.

This finding runs contrary to our hypothesis which posited that lower CR and BR would demonstrate self-regulation and more adverse driving. Instead, our results suggest that older drivers with lower BR and CR are more likely to restrict their driving behaviors compared to their high performer counterparts as demonstrated in over adverse driving, speeding, and trips made during the day. However, low performers also show a higher number of trips taken over time regardless of time, as well as a significant increase in night driving. The increase in night driving may be a result of less traffic or congestion on their roads. With reduced drivers or traffic distractions during nighttime hours, drivers with decreased HV and WBV, and lower CR may feel more comfortable driving. In a recent study that assessed cognitive domain scores and daily driving behavior, individuals with lower attentional control drove less frequently and in smaller proximity to their home but drove more often in their routinely visited areas (Aschenbrenner et al., 2022). Therefore, if individuals perceived night driving as requiring fewer external stimuli, they may be more apt to drive during those times. Previous research investigated the relationship between attentional control and age-related atrophy. Researchers found evidence of decreased responsiveness of cortexes such as dorsolateral prefrontal and parietal regions that are associated with attentional control (Milham et al., 2002). These findings suggest that further investigation is needed to understand CR impact on attentional control and driving behavior changes as associations between cortical function, attentional control, and driving are shown to exist.

As we age, neuronal loss results in atrophy of areas like the hippocampus and cortical regions (O’banion et al., 1994; Morrison and Hof, 1997). Furthermore, results from this study support that BR and CR as proxied by scores on the WRAT 4 may be rather comparable as our results were nearly identical across reserve constructs. This suggests that BR may be more easily assessed through reading fluency tasks. This has been demonstrated in recent research investigating neural efficiency as a predictor of CR and WBV where it was found that CR and WBV can predict neural efficiency (Argiris et al., 2022). Additionally, past research has viewed CR as an index of neuroplasticity (Bartres-Faz and Arenaza-Urquijo, 2011). Due to the complexities of completing MRI in patient population with metal implants or claustrophobia, easily administered tasks of reading fluency may be more efficient.

Past CR and BR research have focused on specific diseases of aging such as Alzheimer’s disease; however longitudinal studies in these pathologies are limited (Groot et al., 2018; Soldan et al., 2020). For example, previous research has shown that individuals in the earliest stages of Alzheimer’s disease (CDR 0.5 and 1) can demonstrate impaired driving in attention-related tasks (Duchek et al., 1998; Stein and Dubinsky, 2011). However, this research was conducted utilizing driving simulators (Stein and Dubinsky, 2011). Additionally, Argiris et al. (2022) investigated behavioral tasks such as reaction time, a large component to daily driving behavior, in a small sample of mixed age groups (young adult and older adult) to identify a new proxy for CR and found that this was positively associated with cortical thickness. While these studies investigate CR from the lens of a simulated IADL, they are limited in their findings as they may not necessarily be generalizable to daily driving which is uncontrolled, dynamically changing, and highly variable. The literature examining CR and BR is unable to speak to possible change over time especially in a cognitively normal older adult sample (Soldan et al., 2020; McQuail et al., 2021). Our present study adds an additional layer to the limited literature by addressing both CR and BR in a cognitively normal sample investigating longitudinal change in a complex IADL with naturalistic data.

The current directions in CR/BR literature have advocated for investigation of more complex and specific proxies to better demonstrate the breadth of reserve (Nilsson and Lövdén, 2018; Stern et al., 2020). Prior research suggests that cognitive tasks are not often associated with BR evincing the need for more sensitive measurement (Neth et al., 2020). Our results indicate that cognitive tasks are not predictive of the difference between groups, with the exception of WBV, providing strong evidence that naturalistic IADLs may be more closely aligned as a suitable proxy for CR than previously used cognitive measures and educational proxies. Due to parallel results between CR and BR in longitudinal changes in driving behavior, future research may also begin to utilize naturalistic driving behavior as a proxy for both CR and BR. However, experts in the field suggest utilizing predictive interaction models between naturalistic driving behavior and brain pathology (Stern et al., 2020).

The results showing a more significant decline in adverse driving behaviors for those with decreased HV and WBV may support the mechanism behind the synaptic pruning theory. This theory suggests that more routine or local processes are completed with greater efficiency due to the elimination of redundant synapses (Stephan et al., 2012; Megias et al., 2018). The elimination of synapses that are redundant does not necessarily infer a lack of learned information but may suggest that more efficient and, quite possibly, stronger synapses are formed for routine or overlearned tasks such as driving. In this study, older drivers with lower brain volume were found to significantly self-regulate their driving behaviors over time which may be attributed to less, but stronger synapses formed due to synaptic pruning. Previous research has shown reduction in brain volume for experts in areas such as playing instruments (Vaguero et al., 2016) or performing ballet (Nigmatullina et al., 2015). The present study was unable to factor in driving history due to possible recall bias which may have further supported the synaptic pruning theory for driving as a highly learned behavior and IADL. Future research should investigate the impact of length of time and frequency of driving history on both CR/BR in relations to driving behaviors.

The present study examined CR and BR in two groups, investigating the low performers (bottom 1/3) with the high performers (top 2/3), however, several limitations exist. It may be important to consider individuals with a range/continuous CR, instead of a dichotomous group (high and low CR). Due to increased participant burden, cost, and MRI radiological considerations was only conducted at baseline. Future research should further investigate BR with additional detail by incorporating longitudinal MRI data. An increased sample size with more diverse representation (race, ethnicity, education) may assist in further generalizability to the larger population since our sample was majority highly educated, non-Hispanic white individuals. An adult’s experience/exposure with driving over many decades may influence the rate of decline over time which was not captured in this study. However, the intraindividual change captured over the data collection period would account for that experience since the older adults are in driving in their own vehicle and home environments. Future research should build on the limited body of literature regarding the area deprivation index (ADI) that is investigating environmental disadvantages across populations. Past work has shown that it may be easier to build and preserve CR in environments fostering physical, social, and cognitively beneficial activities (Vassilaki et al., 2022). Investigating the built and natural environment’s impact on driving and CR could further clarify the relationship between driving behavior changes and CR. Past research has also shown that maintenance of the functional networks associated with an individual’s ability to interpret and respond to external stimuli could preserve driving abilities overtime (Wisch et al., 2022). Additional work could investigate the relationship between CR and functional connectivity to better understand the maintenance of driving ability for the longevity of older adult drivers.

We sought to understand the relationship between reserve on changes in daily driving (an IADL) using a novel naturalistic methodology. The relationship between driving was explored *via* BR, through WBV and HV, and CR, as proxied by the WRAT 4. Older drivers with lower levels of CR and BR demonstrated an increase in idle time but reduced incidents of over speeding and adverse behavior. Additionally, these drivers demonstrated an increase in the variability of start and end time of their trips, but especially showed an increase in night trips. Their high performer CR and BR counterparts are more likely to restrict their driving during all times of the day. Driving remains a complex task that hundreds of millions of drivers safely complete on a daily basis. Changes in CR and BR may presage decline in driving and could be leveraged as screening to better characterize patterns in behavior among older drivers.

## Data availability statement

The raw data supporting the conclusions of this article will be made available by the authors, without undue reservation.

## Ethics statement

The studies involving human participants were reviewed and approved by Washington University Institutional Review Board. The patients/participants provided their written informed consent to participate in this study.

## Author contributions

GB and SM designed the study and wrote the manuscript with contributions from all authors. LC performed the data analysis. All authors contributed to the article and approved the submitted version.

## Funding

This study is supported by the National Institute of Health/National Institute on Aging (grant numbers: AG068183, AG067428, and AG056466) and the BrightFocus Foundation (A2021142S). DC declares that they received additional support from NIA, MoDOT, the Traffic Injury Research Foundation, Medscape, UpToDate, Hoffman La Roche, and GreenValley. The funders were not involved in the study design, collection, analysis, interpretation of data, the writing of this article, or the decision to submit it for publication.

## Conflict of interest

The authors declare that the research was conducted in the absence of any commercial or financial relationships that could be construed as a potential conflict of interest.

## Publisher’s note

All claims expressed in this article are solely those of the authors and do not necessarily represent those of their affiliated organizations, or those of the publisher, the editors and the reviewers. Any product that may be evaluated in this article, or claim that may be made by its manufacturer, is not guaranteed or endorsed by the publisher.

## References

[ref1] Andrews E. C. Westerman S. J. (2012). Age differences in simulated driving performance: compensatory processes. Accid. Anal. Prev. 45, 660–668. doi: 10.1016/j.aap.2011.09.047, PMID: 22269555

[ref2] Argiris G. Stern Y. Habeck C. (2022). Neural similarity across task load relates to cognitive reserve and brain maintenance measures on the letter Sternberg task: A longitudinal study. 10.1007/s11682-022-00746-2 PMC9925407 36484923

[ref3] Armitage S. G. (1946). An analysis of certain psychological tests used for the evaluation of brain injury. Psychol. Monogr. 60, 1–48. doi: 10.1037/h0093567

[ref4] Aschenbrenner A. J. Murphy S. A. Doherty J. M. Johnson A. M. Bayat S. Walker A.. (2022). Neuropsychological correlates of changes in driving behavior among clinically healthy older adults. J. Gerontol.: Seri. B 77, 1769–1778. doi: 10.1093/geronb/gbac101, PMID: 35869666 PMC9535782

[ref5] Babulal G. M. (2022). Predicting driving decline and assessing crash risk in a globally aging population. Arq. Neuropsiquiatr. 80, 1–2. doi: 10.1590/0004-282x-anp-2022-e001, PMID: 35239808 PMC9651509

[ref6] Babulal G. M. Stout S. H. Benzinger T. L. S. Ott B. R. Carr D. B. Webb M.. (2019). A naturalistic study of driving behavior in older adults and preclinical Alzheimer disease: a pilot study. J. Appl. Gerontol. 38, 277–289. doi: 10.1177/0733464817690679, PMID: 28380718 PMC5555816

[ref7] Babulal G. M. Traub C. M. Webb M. Stout S. H. Addison A. Carr D. B.. (2016). Creating a driving profile for older adults using GPS devices and naturalistic driving methodology. F1000Res. 5:2376. doi: 10.12688/f1000research.9608.2, PMID: 27990264 PMC5133689

[ref8] Baker L. M. Laidlaw D. H. Cabeen R. Akbudak E. Conturo T. E. Correia S.. (2017). Cognitive reserve moderates the relationship between neuropsychological performance and white matter fiber bundle length in healthy older adults. Brain Imaging Behav. 11, 632–639. doi: 10.1007/s11682-016-9540-7, PMID: 26961092 PMC7083104

[ref9] Balzarotti S. Biassoni F. Confalonieri F. Meinero C. A. Ciceri M. R. (2021). Cognitive reserve and driving-related cognitive abilities in a sample of oldest old drivers undergoing assessment of fitness to drive. J. Appl. Gerontol. 40, 1758–1767. doi: 10.1177/0733464821994703, PMID: 33645249

[ref10] Bartres-Faz D. Arenaza-Urquijo E. M. (2011). Structural and functional imaging correlates of cognitive and brain reserve hypotheses in healthy and pathological aging. Brain Topogr. 24, 340–357. doi: 10.1007/s10548-011-0195-9, PMID: 21853422

[ref11] Barulli D. Stern Y. (2013). Efficiency, capacity, compensation, maintenance, plasticity: emerging concepts in cognitive reserve. Trends in cognitive sciences, 17, 502-509.Wilkinson, G. S. & Robertson, G. J. 2006. Wide range achievement test (WRAT4). Lutz, FL: Psychological Assessment Resources. 10.1016/j.tics.2013.08.012 PMC3840716 24018144

[ref12] Brickman A. M. Siedlecki K. L. Muraskin J. Manly J. J. Luchsinger J. A. Yeung L. K.. (2011). White matter hyperintensities and cognition: testing the reserve hypothesis. Neurobiol. Aging 32, 1588–1598. doi: 10.1016/j.neurobiolaging.2009.10.013, PMID: 19926168 PMC2891625

[ref13] Buckner R. L. Head D. Parker J. Fotenos A. F. Marcus D. Morris J. C.. (2004). A unified approach for morphometric and functional data analysis in young, old, and demented adults using automated atlas-based head size normalization: reliability and validation against manual measurement of total intracranial volume. NeuroImage 23, 724–738. doi: 10.1016/j.neuroimage.2004.06.018, PMID: 15488422

[ref14] Campbell M. K. Bush T. L. Hale W. E. (1993). Medical conditions associated with driving cessation in community-dwelling, ambulatory elders. J. Gerontol. 48, S230–S234. doi: 10.1093/geronj/48.4.S230, PMID: 8315247

[ref15] Duchek J. M. Hunt L. Ball K. Buckles V. Morris J. C. (1998). Attention and driving performance in Alzheimer's disease. J. Gerontol. Ser. B Psychol. Sci. Soc. Sci. 53, 130–141. 10.1093/geronb/53b.2.p130 9520930

[ref16] Edwards J. D. Lunsman M. Perkins M. Rebok G. W. Roth D. L. (2009). Driving cessation and health trajectories in older adults. J. Gerontol. Ser. A: Biomed. Sci. Med. Sci. 64, 1290–1295. doi: 10.1093/gerona/glp114 PMC2773808 19675177

[ref17] Freeman E. E. Gange S. J. Muñoz B. West S. K. (2006). Driving status and risk of entry into long-term care in older adults. Am. J. Public Health 96, 1254–1259. doi: 10.2105/AJPH.2005.069146, PMID: 16735633 PMC1483865

[ref18] Grober E. Buschke H. Crystal H. Bang S. Dresner R. (1988). Screening for dementia by memory testing. Neurology 38, 900–903. doi: 10.1212/WNL.38.6.900 3368071

[ref19] Groot C. Van Loenhoud A. C. Barkhof F. Van Berckel B. N. Koene T. Teunissen C. C.. (2018). Differential effects of cognitive reserve and brain reserve on cognition in Alzheimer disease. Neurology 90, e149–e156. doi: 10.1212/WNL.0000000000004802, PMID: 29237798

[ref01] Koenig L. N. Lamontagne P. Glasser M. F. Bateman R. Holtzman D. Yakushev I.. (2022). Regional age-related atrophy after screening for preclinical Alzheimer disease. Neurobiol. Aging 109, 43–51. doi: 10.1016/j.neurobiolaging.2021.09.010 34655980 PMC9009406

[ref20] Koenig L. N. Mccue L. M. Grant E. Massoumzadeh P. Roe C. M. Xiong C.. (2021). Lack of association between acute stroke, post-stroke dementia, race, and beta-amyloid status. Neuroimage Clin. 29:102553. doi: 10.1016/j.nicl.2020.102553, PMID: 33524806 PMC7848631

[ref22] Mcquail J. A. Dunn A. R. Stern Y. Barnes C. A. Kempermann G. Rapp P. R.. (2021). Cognitive reserve in model systems for mechanistic discovery: the importance of longitudinal studies. Front. Aging Neurosci. 12:607685. doi: 10.3389/fnagi.2020.607685, PMID: 33551788 PMC7859530

[ref23] Megias A. Petrova D. Navas J. F. Candido A. Maldonado A. Catena A. (2018). Neuroanatomical variations as a function of experience in a complex daily task: a VBM and DTI study on driving experience. Brain Imaging Behav. 12, 653–662. doi: 10.1007/s11682-017-9725-8, PMID: 28447245

[ref24] Milham M. P. Erickson K. I. Banich M. T. Kramer A. F. Webb A. Wszalek T.. (2002). Attentional control in the aging brain: insights from an fMRI study of the stroop task. Brain Cogn. 49, 277–296. doi: 10.1006/brcg.2001.1501, PMID: 12139955

[ref25] Morris J. C. (1993). The clinical dementia rating (CDR): current version and scoring rules. Neurology 43, 2412–2414. doi: 10.1212/WNL.43.11.2412-a, PMID: 8232972

[ref26] Morrison J. H. Hof P. R. (1997). Life and death of neurons in the aging brain. Science 278, 412–419. doi: 10.1126/science.278.5337.412 9334292

[ref27] Neth B. J. Graff-Radford J. Mielke M. M. Przybelski S. A. Lesnick T. G. Schwarz C. G.. (2020). Relationship between risk factors and brain reserve in late middle age: implications for cognitive aging. Front. Aging Neurosci. 11:355. doi: 10.3389/fnagi.2019.00355, PMID: 31998113 PMC6962238

[ref28] Nigmatullina Y. Hellyer P. J. Nachey P. Sharp D. J. Seemungal B. M. (2015). The neuroanatomical correlates of training-related perceptuo-reflex uncoupling in dancers. Cereb. Cortex 25, 554–562. doi: 10.1093/cercor/bht266, PMID: 24072889 PMC4380084

[ref29] Nilsson J. Lövdén M. (2018). Naming is not explaining: future directions for the “cognitive reserve” and “brain maintenance” theories. Alzheimers Res. Ther. 10, 1–7. doi: 10.1186/s13195-018-0365-z 29609632 PMC5879611

[ref30] O’banion M. K. Coleman P. D. Callahan L. M. (1994). Regional neuronal loss in aging and Alzheimer’s disease: a brief review. Semin. Neurol. 6, 307–314. doi: 10.1006/smns.1994.1039

[ref31] O’neill D. Bruce I. Kirby M. Lawlor B. (2000). Older drivers, driving practices and health issues. Clin. Gerontol. 22, 47–54.

[ref32] Pollack M. E. (2005). Intelligent technology for an aging population: the use of AI to assist elders with cognitive impairment. AI Mag. 26, 9–9. doi: 10.1609/aimag.v26i2.1810

[ref33] Qin W. Xiang X. Taylor H. (2020). Driving cessation and social isolation in older adults. J. Aging Health 32, 962–971. doi: 10.1177/0898264319870400, PMID: 31431103 PMC7901288

[ref34] Roe C. M. Babulal G. M. Head D. M. Stout S. H. Vernon E. K. Ghoshal N.. (2017a). Preclinical Alzheimer's disease and longitudinal driving decline. Alzheimer's Dementia: Trans. Res. Clin. Interv. 3, 74–82. doi: 10.1016/j.trci.2016.11.006, PMID: 28435853 PMC5396459

[ref35] Roe C. M. Barco P. P. Head D. M. Ghoshal N. Selsor N. Babulal G. M.. (2017b). Amyloid imaging, cerebrospinal fluid biomarkers predict driving performance among cognitively normal individuals. Alzheimer Dis. Assoc. Disord. 31, 69–72. doi: 10.1097/WAD.0000000000000154, PMID: 27128959 PMC5085874

[ref36] Roe C. M. Rosnick C. B. Colletta A. Babulal G. M. (2021). Reaction to a pandemic: social distancing and driving among older adults during COVID-19. J. Appl. Gerontol. 40, 263–267. doi: 10.1177/0733464820966516, PMID: 33554720 PMC7871015

[ref37] Roe C. M. Stout S. H. Rajasekar G. Ances B. M. Jones J. M. Head D.. (2019). A 2.5-year longitudinal assessment of naturalistic driving in preclinical Alzheimer's disease. J. Alzheimers Dis. 68, 1625–1633. doi: 10.3233/JAD-181242, PMID: 30958365 PMC6488385

[ref38] Rosen H. E. Bari I. Paichadze N. Peden M. Khayesi M. Monclús J. Hyder A. A. (2022). Global road safety 2010–18: an analysis of global status reports. Injury. 10.1016/j.injury.2022.07.030 35906119

[ref39] Siedlecki K. L. Stern Y. Reuben A. Sacco R. L. Elkind M. S. Wright C. B. (2009). Construct validity of cognitive reserve in a multiethnic cohort: the northern Manhattan study. J. Int. Neuropsychol. Soc. 15, 558–569. doi: 10.1017/S1355617709090857 19573274 PMC2803322

[ref40] Soldan A. Pettigrew C. Albert M. (2020). Cognitive reserve from the perspective of preclinical Alzheimer disease: 2020 update. Clin. Geriatr. Med. 36, 247–263. doi: 10.1016/j.cger.2019.11.006, PMID: 32222300 PMC7837205

[ref41] Stein A. C. Dubinsky R. M. (2011). Driving simulator performance in patients with possible and probable Alzheimer’s disease. Annals of advances in automotive medicine/annual scientific conference. Association for the Advancement of Automotive Medicine, 325. PMC3256816 22105407

[ref42] Stephan A. H. Barres B. A. Stevens B. (2012). The complement system: an unexpected role in synaptic pruning during development and disease. Annu. Rev. Neurosci. 35, 369–389. doi: 10.1146/annurev-neuro-061010-113810, PMID: 22715882

[ref43] Stern Y. Arenaza-Urquijo E. M. Bartrés-Faz D. Belleville S. Cantilon M. Chetelat G.. (2020). Whitepaper: defining and investigating cognitive reserve, brain reserve, and brain maintenance. Alzheimers Dement. 16, 1305–1311. doi: 10.1016/j.jalz.2018.07.219, PMID: 30222945 PMC6417987

[ref44] Suchy Y. Kraybill M. L. Franchow E. (2011). Instrumental activities of daily living among community-dwelling older adults: discrepancies between self-report and performance are mediated by cognitive reserve. J. Clin. Exp. Neuropsychol. 33, 92–100. doi: 10.1080/13803395.2010.493148, PMID: 20623400

[ref45] Tombaugh T. N. Kozak J. Rees L. (1999). Normative data stratified by age and education for two measures of verbal fluency: FAS and animal naming. Arch. Clin. Neuropsychol. 14, 167–177. PMID: 14590600

[ref46] Tucker A. M. Stern Y. (2011). Cognitive reserve in aging. Curr. Alzheimer Res. 8, 354–360. doi: 10.2174/156720511795745320, PMID: 21222591 PMC3135666

[ref47] Vaguero L. Hartmann K. Ripolles P. Rojo N. Sierpowska J. Francois C.. (2016). Structural neuroplasticity in expert pianists depends on the age of musical training onset. NeuroImage 126, 106–119. doi: 10.1016/j.neuroimage.2015.11.008, PMID: 26584868

[ref48] Vassilaki M. Aakre J. Castillo A. Chamberlain A. Wilson P. Kremers W. Mielke M. Geda Y. Machulda M. Alhurani R. (2022). Area deprivation index and progression to dementia (S2.007). AAN Enterprises.

[ref02] Wilkinson G. S. Robertson G. J. (2006). Wide range achievement test (WRAT4). Lutz, FL: Psychological Assessment Resources.

[ref49] Wisch J. K. Roe C. M. Babulal G. M. Metcalf N. Johnson A. M. Murphy S.. (2022). Naturalistic driving measures of route selection associate with resting state networks in older adults. Sci. Rep. 12, 1–8. doi: 10.1038/s41598-022-09919-x 35443765 PMC9021301

